# Discovering Cancer Subtypes via an Accurate Fusion Strategy on Multiple Profile Data

**DOI:** 10.3389/fgene.2019.00020

**Published:** 2019-02-05

**Authors:** Limin Jiang, Yongkang Xiao, Yijie Ding, Jijun Tang, Fei Guo

**Affiliations:** ^1^School of Computer Science and Technology, College of Intelligence and Computing, Tianjin University, Tianjin, China; ^2^School of Chemical Engineering and Technology, Tianjin University, Tianjin, China; ^3^School of Electronic and Information Engineering, Suzhou University of Science and Technology, Suzhou, China; ^4^Department of Computer Science and Engineering, University of South Carolina, Columbia, SC, United States

**Keywords:** cancer subtypes prediction, similarity kernel fusion, spectral clustering, sparse matrix, The Cancer Genome Atlas

## Abstract

Discovering cancer subtypes is useful for guiding clinical treatment of multiple cancers. Progressive profile technologies for tissue have accumulated diverse types of data. Based on these types of expression data, various computational methods have been proposed to predict cancer subtypes. It is crucial to study how to better integrate these multiple profiles of data. In this paper, we collect multiple profiles of data for five cancers on The Cancer Genome Atlas (TCGA). Then, we construct three similarity kernels for all patients of the same cancer by gene expression, miRNA expression and isoform expression data. We also propose a novel unsupervised multiple kernel fusion method, Similarity Kernel Fusion (SKF), in order to integrate three similarity kernels into one combined kernel. Finally, we make use of spectral clustering on the integrated kernel to predict cancer subtypes. In the experimental results, the *P*-values from the Cox regression model and survival curve analysis can be used to evaluate the performance of predicted subtypes on three datasets. Our kernel fusion method, SKF, has outstanding performance compared with single kernel and other multiple kernel fusion strategies. It demonstrates that our method can accurately identify more accurate subtypes on various kinds of cancers. Our cancer subtype prediction method can identify essential genes and biomarkers for disease diagnosis and prognosis, and we also discuss the possible side effects of therapies and treatment.

## 1. Introduction

Cancer is a heterogeneous disease caused by chemical, physical, or genetic factors (Mager, 2006; Liu and Chu, 2014). The development of high-throughput genome analysis techniques on the research of cancer subtypes plays an important role in the analysis and clinical treatment of various kinds of cancers (Kruijf et al., 2013; Prat et al., 2015; Thanki et al., 2017). In recent years, much expression data, including genomes, transcriptome and epigenomes, has accumulated and been stored in various databases. The Cancer Genome Atlas (TCGA) (Katarzyna et al., 2015) is a large-scale project including over 34 cancers and 15 expression data sets. We can conveniently obtain genome-scale molecular data, which contributes to the development of computational methods for discovering cancer subtypes.

Until now, massive computational methods were proposed to discover cancer subtypes. Some methods are based on single expression data, including gene expression data (Nguyen and Rocke, 2002; Brunet et al., 2004; Finnegan and Carey, 2007; Teschendorff et al., 2007) and copy number (Wong et al., 2012) and DNA methylation (Zhang et al., 2017). Gao and Church (2005) employed sparse non-negative matrix factorization (SNMF) and gene expression data to identify subtypes of three cancers. Also, various kinds of expression data (Wei et al., 2017, 2018a,b) and several types of similarity strategies (Zeng et al., 2016; Ding et al., 2017a,b; Pan et al., 2017, 2018; Guo F. et al., 2018; Song et al., 2018) can be applied in many other biological prediction problems.

Generally, we desire a comprehensive view of one disease with a cohort of patients. We cannot analyze just one kind of data, but must separately abstract information from different types of data (Xu et al., 2017). Therefore, many methods improve the robustness of clustering by focusing on data processing (Ren et al., 2015). Wang et al. (2014) proposed the Similarity Network Fusion (SNF) approach for accurately clustering caner subtypes. This method first collects three types of genome-wide data including gene, methylation and miRNA expression. Then, it constructs the networks of samples (e.g., patients) by using three types of expression data, and fuses these networks into one network by using SNF representing the full spectrum of underlying data. Finally, it employs spectral clustering on an integrated network to predict caner subtypes. Ma and Zhang (2017) developed an improved SNF, Affinity Network Fusion (ANF), to integrate multiple similarity networks. Xu et al. (2016) proposed Weighted Similarity Network Fusion (WSNF) to identify cancer subtypes. This method constructs similarity of patients by integrating associations between miRNA, mRNA, and transcription factors. It is applied to two cancer types to demonstrate performance.

Furthermore, the effective models of clustering that we usually use have strong data sensitivity, such as k-means and hierarchical clustering. Today, many clustering methods have been developed to identify cancer subtypes. Le et al. (2016) developed the SRF algorithm, which identifies subtypes by combining mutational and expression information. It diffuses mutation information over an interaction network on the basis of each sample and eliminates scale differences by applying a rank-based transformation based on mutation and expression data. Then, rank matrix factorization is used to jointly factorize the transformed data into a number of ranked factors, and the subtypes are defined as the combination of ranked factors. This method obtains excellent performance, but some of the patients cannot be identified. Shen et al. (2009) proposed the iCluster method, which is based on the Gaussian latent variable model, to discover caner subtypes. This method was tested on breast cancer and lung cancer by using copy number and gene expression data types. Speicher and Pfeifer (2015) pointed out that iCluster has high computational complexity and proposed a dimensionality reduction method to integrate multiple similarity kernels. This method is evaluated by using five cancer types. Ge et al. (2017) developed the Scluster method, which integrates different types of data and maps them into an effective low-dimensional subspace. First, Scluster uses adaptive sparse reduced-rank regression (S-rrr) to map the original data into the principal subspaces. Next, a fused patient-by-patient network is abstracted for these subgroups by a scaled exponential similarity kernel method. It can then obtain the cancer subtypes by spectral clustering.

In this paper, we first collect multiple profile data on The Cancer Genome Atlas (TCGA), including five cancers (lung cancer, kidney cancer, stomach cancer, breast cancer, and colon cancer) and their three types of expression data (gene expression, isoform expression, and miRNA expression). Then, we construct three similarity kernels for all patients of the same cancer by using the three types of expression data. We then propose a novel unsupervised multiple kernel fusion method, Similarity Kernel Fusion (SKF), in order to integrate three similarity kernels into one combined kernel. Compared with SNF, SKF not only keeps the original information of each type of similarity kernel, but also gets rid of the noise in the integrated kernel. Finally, we make use of spectral clustering on the integrated kernel to predict cancer subtypes. To test the effectiveness and robustness of this novel approach, *P*- values from a Cox regression model and survival curve analysis can be used to evaluate the performance of our method on cancer subtype prediction. We compare the integrated kernel with the single kernel and other fusion methods, and also analyze the survival curve of the clinical data.

## 2. Materials and Methods

**Figure 1:**
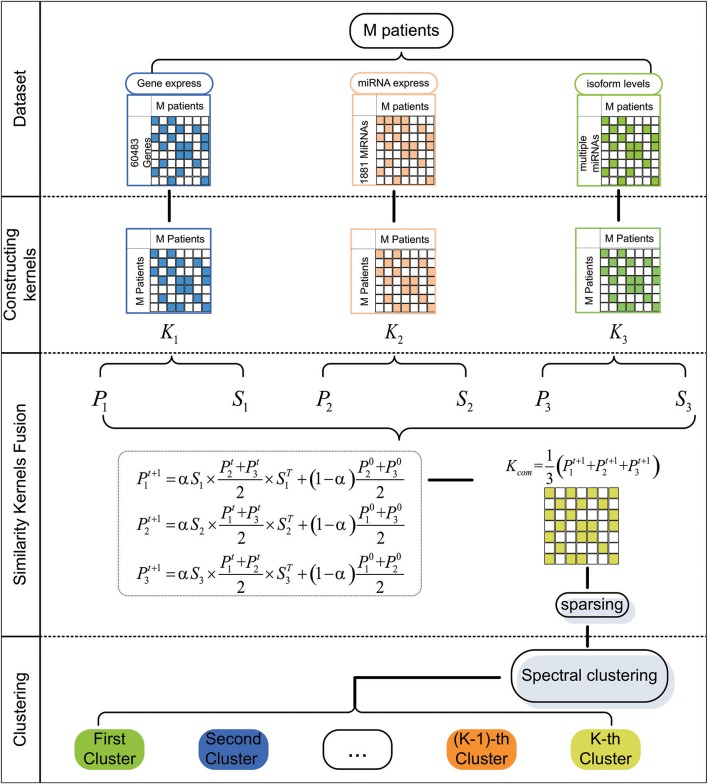
The flowchart of our method for discovering cancer subtype.

In this paper, we first extract five cancer datasets from The Cancer Genome Atlas (TCGA). For a particular cancer, we construct three patient similarity kernels by using the expression data. Then, we combine these similarity kernels into one similarity kernel by using Similarity Kernel Fusion (SKF). Finally, we employ spectral clustering on the integrated kernel to divide all patients into multiple clusters. The flowchart of our method is shown in Figure 1.

### 2.1. Dataset

We collect five cancer datasets from the TCGA website, including stomach cancer, lung cancer, kidney cancer, breast cancer, and colon cancer. For each cancer, we extract three kinds of expression data respectively, including gene expression, miRNA expression, and isoform level. Our dataset is denoted as Dataset No.1 in this paper. In addition, we employ anther dataset to evaluate the performance of our method. The second dataset is provided in Wang et al. (2014), which includes lung cancer, kidney cancer, breast cancer, colon cancer, and glioblastoma multiforme (GBM). For each tumor, gene expression, methylation expression, and miRNA expression from TCGA are used to analyze cancer subtypes. We denote this dataset as Dataset No.2. Since genes could be categorized into multiple groups, we selected 18222 coding genes from Dataset No.1, formed as Dataset No.3 . A summary of the three datasets is shown in Table 1. It is clear that Dataset No.1 and Dataset No.3 have more patients and expression factors than Dataset No.2 .

**Table 1:** Description of three datasets from TCGA.

	**Diseases**	**Patients**	**Genes**	**Isoform**	**miRNAs**
No.1 Dataset	Breast	1071	60483	183	1881
	Colon	426	60483	186	1881
	Kidney	868	60483	176	1881
	Lung	981	60483	174	1881
	Stomach	377	60483	211	1881
	**Diseases**	**Patients**	**Genes**	**CpG sites**	**miRNAs**
No.2 Dataset	Breast	105	17814	23094	354
	Colon	92	17814	23088	312
	Kidney	122	17899	24960	329
	Lung	106	12042	23074	352
	GBM	215	12042	1305	534
	**Diseases**	**Patients**	**Genes**	**Isoform**	**miRNAs**
No.3 Dataset	Breast	1071	18222	183	1881
	Colon	426	18222	186	1881
	Kidney	868	18222	176	1881
	Lung	981	18222	174	1881
	Stomach	377	18222	211	1881

### 2.2. Similarity Kernel Construction

A special expression dataset is denoted as *E*∈*R*^*n*×*m*^, where *m* is the number of expression factors and *n* is the number of patients. We first normalize *E* by using Equation (1).

(1) $$\begin{array}{r} x^{\prime }=\frac{x-\bar{X}}{S} \end{array}$$

where *x* is an element of *E*, x′ is corresponding elements of *E* after standardization, $\bar{X}$ is the mean of *E* and *S* is standard deviation of *E*. And, we denote normalized expression data as *E*′.

Based on the processed expression data *E*′, we construct similarity kernel *K*∈*R*^*m*×*m*^ for patients. Here, the similarity between two patients is defined as Equation (2) (Chen et al., 2018b; Zhao et al., 2018a,b).

(2) $$\begin{array}{r} K_{i,j}=\sqrt{{\left(e_{i}-e_{j}\right)}^{T}\left(e_{i}-e_{j}\right)} \end{array}$$

where *K*_*i,j*_ is the similarity between *i*-th patient and *j*-th patient, $e_{i}\in R^{n\times 1}$ and $e_{j}\in R^{n\times 1}$ is *i*-th column and *j*-th column of *E*′, respectively.

Finally, we get three similarity kernels for a special disease, including similarity kernel $K_{1}\in R^{m\times m}$ by using gene expression, similarity kernel $K_{2}\in R^{m\times m}$ by using miRNA expression, and similarity kernel $K_{3}\in R^{m\times m}$ by using isoform expression.

### 2.3. Similarity Kernel Fusion

We constructed three similarity kernels for patients in the above section. We propose Similarity Kernel Fusion (SKF) to combine these kernels into one kernel K^*^∈*R*^*m*×*m*^. First, we construct two kernels *P*∈*R*^*m*×*m*^ and *S*∈*R*^*m*×*m*^ for each similarity kernel by using Equations (3, 4), where *P* is a normalized kernel and *S* is a sparse kernel that eliminates weak similarity.

(3) $$P(i,j)=\frac{K_{i,j}}{\sum_{k=1}^{m}K_{k,j}}$$

where *P* satisfies $\sum_{k=1}^{m}P(k,j)=1$.

(4) $$S(i,j)=\{\begin{array}{cc} 0 & if\;j\notin N_{i}\\ \frac{K_{i,j}}{\sum_{k\in N_{i}}K_{i,k}} & if\;j\in N_{i} \end{array}$$

where *S* satisfies $\sum_{k=1}^{m}S(i,j)=1$; *N*_*i*_ is a set of all neighbors of the *i*-th patient, including itself.

Second, we discover more information by using multiple iterations as Equation (5).

(5) $$P_{l}^{t+1}=\alpha (S_{l}\times \frac{\sum_{r\neq l}P_{r}^{t}}{2}\times S_{l}^{t})+(1-\alpha )(\frac{\sum_{r\neq l}P_{r}^{0}}{2})$$

where $P_{l}^{t}$ (*l* = 1, 2, 3) is the status of the *l*-th kernel after *t* iterations, α is a coefficient and satisfies α∈[0, 1], $P_{r}^{0}$ (*r* = 1, 2, 3) represents the initial status of *P*_*r*_.

After *t* + 1 iterations, the overall kernel can be computed as Equation (6).

(6) $$K_{com}=\frac{1}{3}\sum_{l=1}^{3}P_{l}^{t+1}$$

Finally, based on the integrated kernel, we construct a weight matrix to eliminate noise in the integrated kernel as Equation (7).

(7) $$w(i,j)=\{\begin{array}{cc} 1 & if\;j\in N_{i}\;\cap \;i\in N_{j}\\ 0 & if\;j\notin N_{i}\;\cap \;i\notin N_{j}\\ 0.5 & otherwise \end{array}$$

where *N*_*i*_ is a set of all neighbors of the *i*-th patient, including itself, and *N*_*j*_ is a set of all neighbors of the *j*-th patient, including itself.

The final similarity kernel can be obtained as Equation (8).

(8) $$\begin{array}{r} K^{*}=w◦K_{com} \end{array}$$

where K^*^ is the final integrated similarity kernel by using SKF.

### 2.4. Mining Subtypes Using Spectral Clustering

In this section, we employ spectral clustering (Ng et al., 2001) on the integrated similarity kernel to divide all patients into multiple clusters. Many previous studies, including CSPRV (Guo Y. et al., 2018), Scluster (Ge et al., 2017), and SNF(Wang et al., 2014), have constructed similarity kernels for patients and used spectral clustering to discover cancer subtypes. These methods have achieved excellent performance by using spectral clustering. Additionally, Luxburg (2007) have pointed out that spectral clustering is effective in capturing the global structure of the graph. Therefore, we use spectral clustering to identify cancer subtypes. Then, we will introduce the processes of spectral clustering in detail. We define a matrix *Y*∈{0, 1}^*k*×*n*^ to represent the result of a cluster, where *Y*(*i, j*) = 1 if patient *p*_*j*_ belongs to *i*-th cluster, otherwise *Y*(*i, j*) = 0. We also use Equation (9) as the optimal question to solve *Y*.

(9) $$\begin{array}{l} \underset{Q\in R^{k\times n}}{\min}Trace(Q^{T}L^{+}Q)\\ \;s.t.Q^{T}Q=I \end{array}$$

where $Q=Y{\left(Y^{\prime }Y\right)}^{-\frac{1}{2}}$, $L^{+}=I-D^{-\frac{1}{2}}K^{*}D^{-\frac{1}{2}}$, *D* is a diagonal matrix whose diagonal element is the sum of the row elements of K^*^.

## 3. Results

In this section, we discuss the performance of our method in a variety of ways. First, we introduce an evaluation criteria and a verification method that are used to evaluate the performance significance of the cancer subtype predictions. Second, we analyze the performance of SKF with different parameters α on Dataset No.1 . Third, we discuss the performance of SKF on the three datasets. Fourth, we compare SKF with two other fusion methods on the three datasets. Finally, we analyze the survival probability curves of the predicted subtypes for four cancers.

### 3.1. Evaluation Criteria and Verification Method

In this paper, we employ the *P*-value from the Cox regression model to evaluate the performance of our method, where a lower *P*-value indicates higher significance for performance. When the *P*-value is less than 0.05, it is of significance to the performance of the model. When the *P*-value is less than 0.01, the performance of the model is highly significant. Here, we use 0.05 as the threshold for significance. The meaning of the *P*-value is significance in the difference of survival profiles between cancer subtypes. Moreover, we also use survival analysis to evaluate the performance of the clustering results. The survival curve represents the change in survival rate over time, and it is a monotone decreasing curve without any fluctuation. In the survival curve, we can find that different subtypes have different survival rates. We can analyze some subtypes that have a higher risk of death.

### 3.2. Parameter Selection for SKF

**Figure 2:**
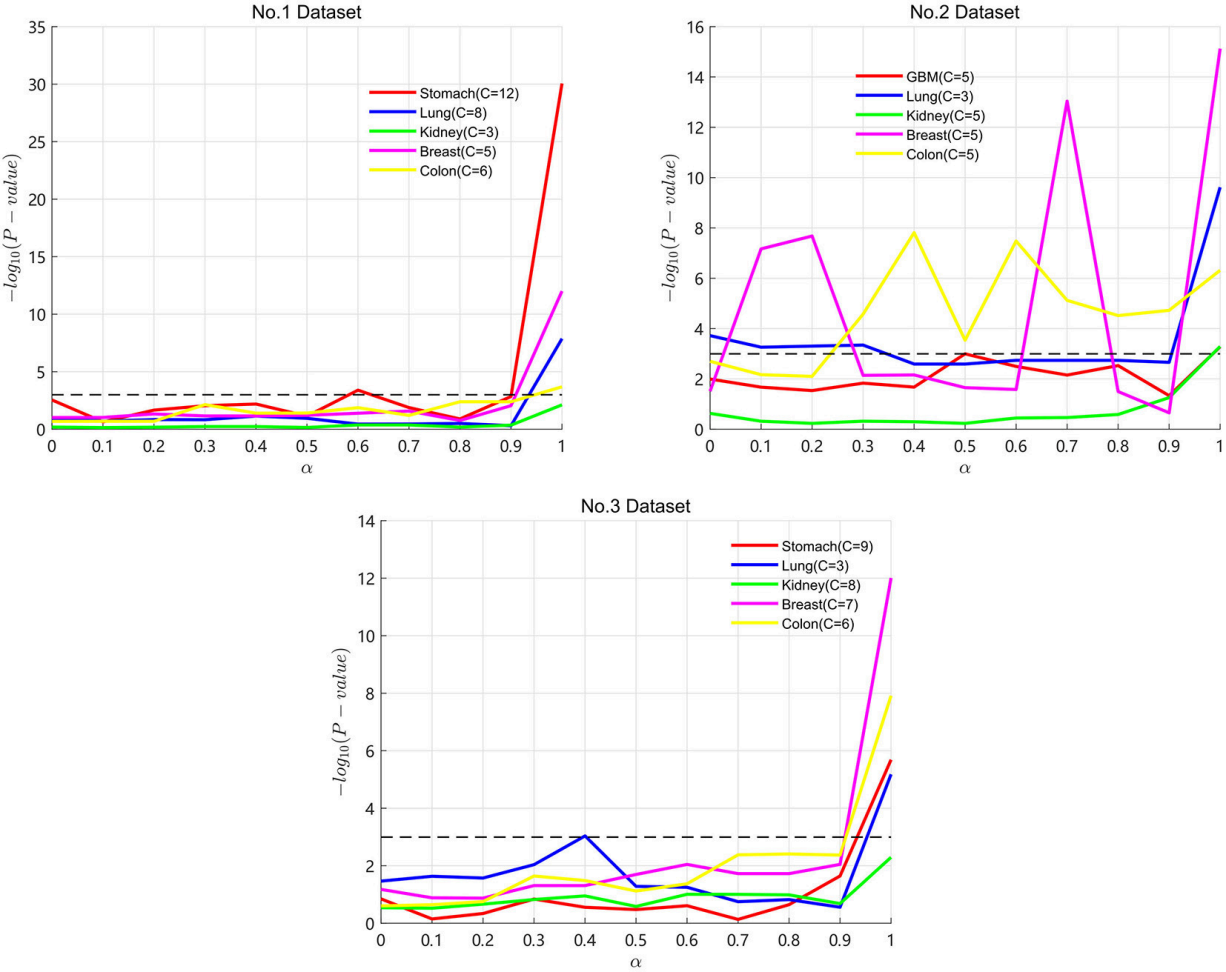
Results of SKF with α on three datasets.

Particularly, α is an important parameter in the process of SKF. A lower α value represents keeping more initial information in the integrated kernel. A higher α value represents keeping more information after multiple iterations. In the three datasets, we take α from 0 to 1 with a step of 0.1 to find the optimal α for the five cancers. Results are shown in Figure 2, with the X axis representing the α value and the Y axis representing the −log_10_(*P*_*value*_). A lower *P*-value is represented by a higher value of −log_10_(*P*_*value*_). In Figure 2, the *P*-value maintains clear fluctuation in the range between 0 and 1. It demonstrates that SKF is sensitive to changes in α. We get the optimal *P*-value when α is equal to 1 for the four cancers except lung cancer on Dataset No.3. From the results of Dataset No.2, we can see that keeping more initial information is necessary for many of the datasets.

### 3.3. Performance of SKF in Difference Datasets

In this paper, we obtain Dataset No.1 from TCGA. For a specific disease, we extract all 60483 gene expression data points on Dataset No.1 . We employ the three datasets to evaluate the performance of SKF. For each dataset, we compare the performance of SKF with single kernel by using the optimal number of clusters. In Table 2, we can see that SKF achieves outstanding performance compared with single kernel in 12 cases. We also find that the same kernels with different numbers of clusters have different *P*-values. Therefore, we need to adjust the number of clusters to obtain optimal clustering results. Although *P*-values do not achieve significant performance for GBM cancer in Dataset No.2 or Kidney cancer in Datasets No.2 and No.3 after SKF, these *P*-values get remarkable promotion compared to single kernel. Moreover, it is clear that the *P*-value of Dataset No.3 is better than that in Dataset No.1 , which shows that coding genes play an important role in the clustering of cancer subtypes.

**Table 2:** Comparison results between SKF and single kernel on three datasets.

**Datasets**	**Cancers**	**Gene**	**miRNA**	**Isoform**	**SKF**
		**expression**	**expression**	**expression**	
Dataset No.1	Stomach (C=12)	0.703	0.027	0.548	8.86 × 10^−14^
	Lung (C = 8)	0.621	0.137	0.829	3.81 × 10^−4^
	Kidney (C = 3)	0.228	0.642	0.358	0.120
	Breast (C = 5)	0.516	0.281	0.281	9.79 × 10^−6^
	Colon (C = 6)	0.045	0.726	0.133	0.025
	**Cancers**	**Gene**	**DNA**	**miRNA**	**SKF**
		**expression**	**methylation**	**expression**	
Dataset No.2	GBM (C = 5)	0.159	0.001	0.436	0.037
	Lung (C = 3)	8.25 × 10^−4^	0.009	0.289	6.66 × 10^−5^
	Kidney (C = 5)	0.0177	0.467	0.368	0.0372
	Breast (C = 5)	0.009	0.00164	1.38 × 10^−4^	2.7 × 10^−7^
	Colon (C = 5)	0.587	0.084	0.702	1.81 × 10^−3^
	**Cancers**	**Gene**	**miRNA**	**Isoform**	**SKF**
		**expression**	**expression**	**expression**	
Dataset No.3	Stomach (C = 9)	0.0538	0.438	0.621	0.003
	Lung (C = 3)	0.352	0.171	0.398	0.005
	Kidney (C = 8)	0.048	0.0018	0.779	0.101
	Breast (C = 7)	0.597	0.0343	0.864 × 10^−8^	1.06 × 10^−34^
	Colon (C = 7)	0.0465	0.626	0.134	3.66 × 10^−4^

### 3.4. Comparing With Other Fusion Methods

**Figure 3:**
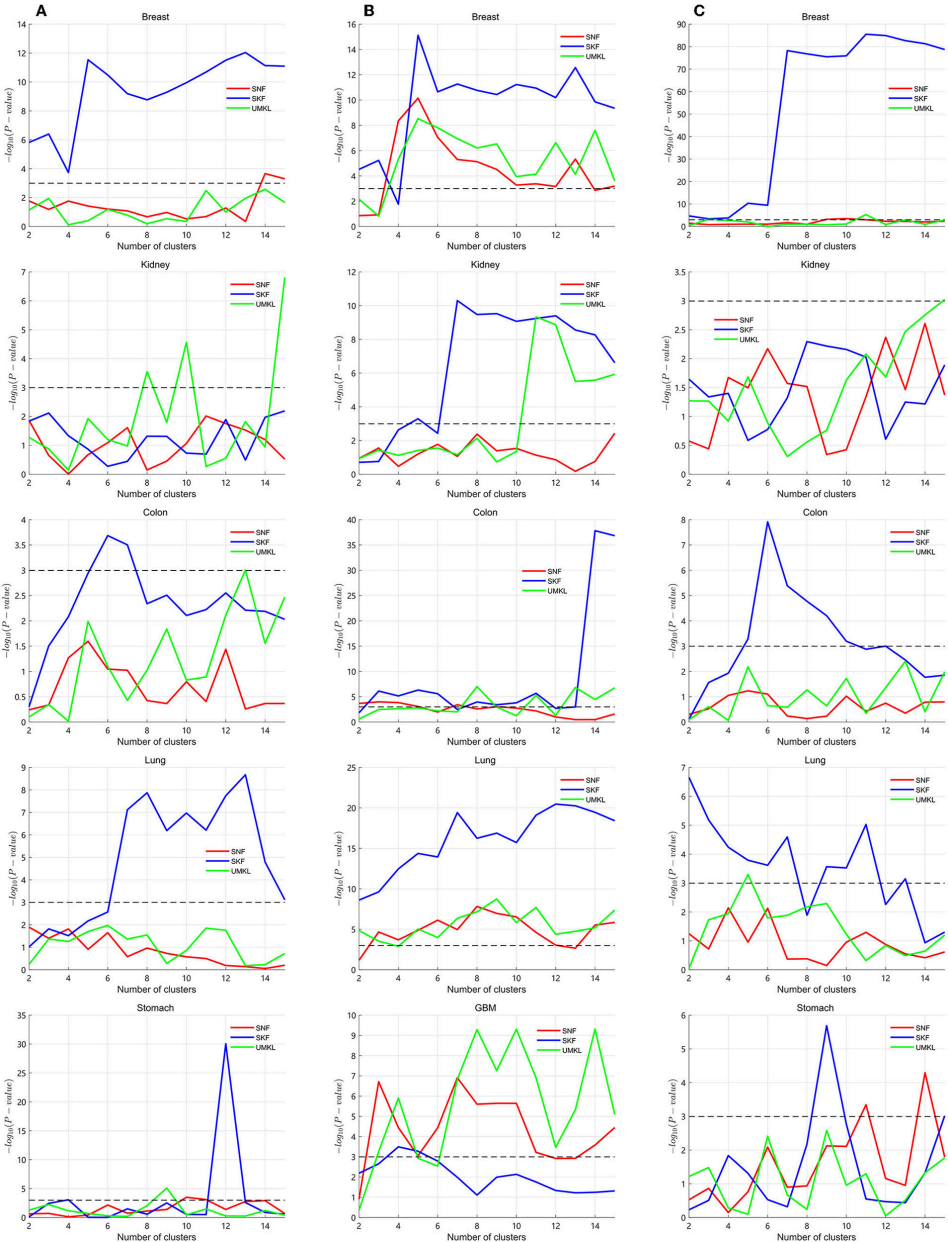
Calculating *P*-values of SKF, SNF, and UMKL with different number of clusters. **(A)** Results of Dataset No.1. **(B)** Results of Dataset No.2. **(C)** Results of Dataset No.3.

Several multiple kernel fusion strategies have been developed, including similarity network fusion (SNF) (Wang et al., 2014) and unsupervised multiple kernel learning (UMKL) (Mariette and Villavialaneix, 2018). We compared the performance of SKF with these two strategies to find better subtypes for a particular cancer. We tested the three strategies on the three datasets to compare the performance of different fusion methods. All results are found in the Supplementary Table 1. The graphical results are shown in Figure 3, with the X axis representing the number of clusters and the Y axis representing the value of −log_10_(*P*_*value*_). The blue lines represent the change of SKF, the red lines represent the change of SNF, the green lines represent the change of UMKL and the black dashed lines show the *P*-value equal to 0.05. In Figure 3, we find that SKF achieved a remarkable level of performance for the clustering of breast and colon cancer subtypes in the three datasets. Additionally, SKF achieved better performance than other kernel fusion strategies for the clustering of lung cancer subtypes in Datasets No.1 and No.3. We also found that SNF performed well for the clustering of kidney cancer subtypes in the three datasets and UMKL reached the best level of performance for the clustering of lung cancer subtypes in Dataset No.2 and stomach cancer subtypes in Dataset No.1. It demonstrates that SKF obtained a significant level performance for discovering subtypes of a particular cancer, and also that the cluster results can be used for guiding clinical treatment.

### 3.5. Survival Analysis

**Figure 4:**
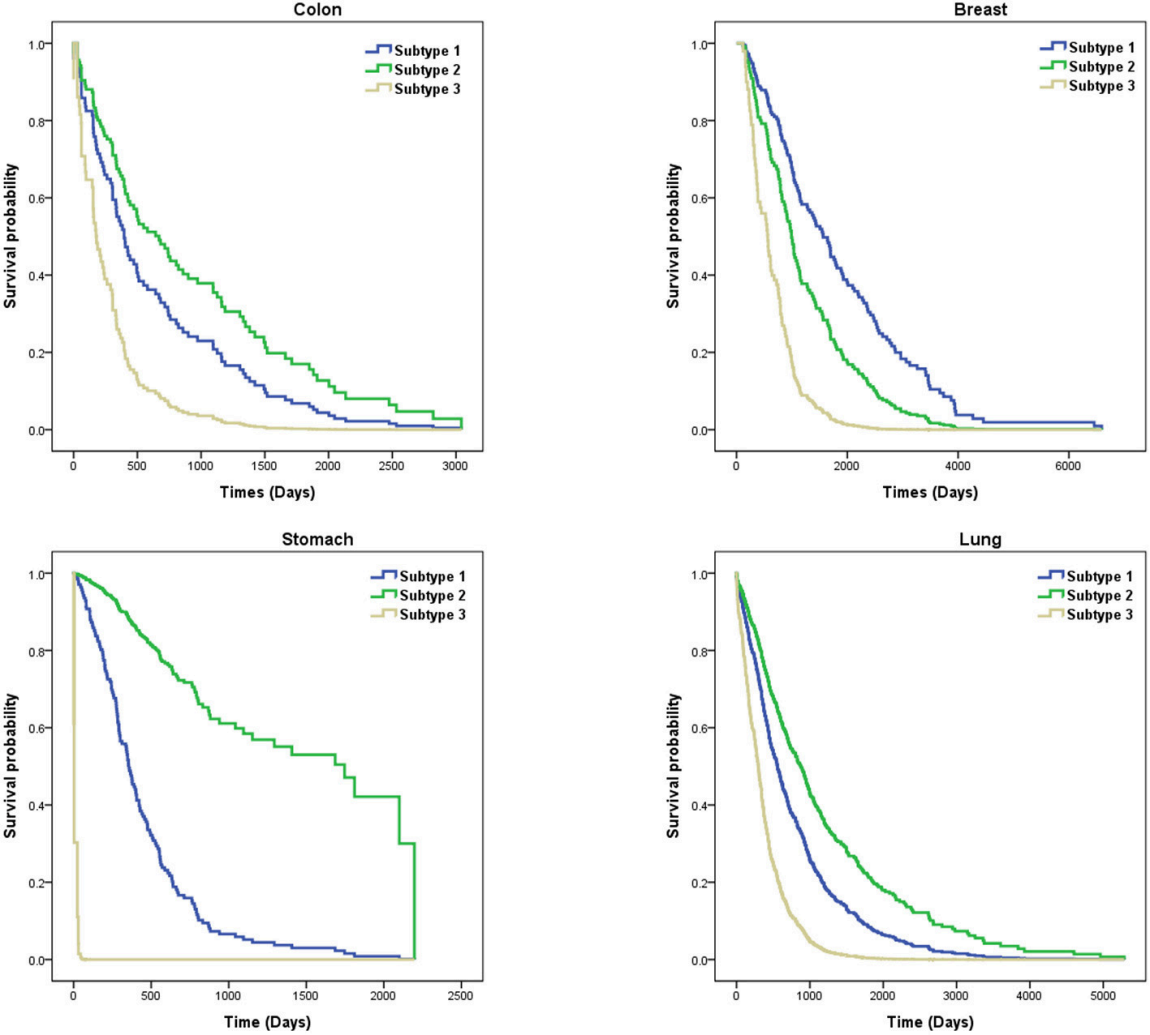
Survival curves of subtypes for four cancers.

In this paper, we analyzed the performance of SKF based on six cancers, including breast, lung, kidney, colon, stomach, and GBM cancers. However, since the *P*-values for the clustering of kidney and GBM cancer subtypes were larger than 0.05, we showed survival probability curves for the four other cancers. We analyzed these cancer subtypes by using Dataset No.3 . In Figure 4, we find that subtype 3 for stomach cancer has a higher death rate. These patients with subtype 3 need more attention to be paid to them. The average survival time of subtype 2 for colon cancer is longer than the other subtypes. Similarly, subtype 3 for other cancers tends to be more aggressive than other subtypes. We also found that the average survival time for breast cancer and lung cancer are longer than for stomach and colon cancer. It demonstrates that the cluster results of SKF can be used to guide clinical treatment.

## 4. Conclusions

In this paper, we proposed an accurate model for predicting cancer subtypes. First, we extracted a novel dataset with three expression data types (gene expression, miRNA expression, and isoform expression) and five cancers (breast, lung, kidney, colon, and stomach cancers) from the TCGA website. Second, we constructed three similarity kernels by using the three types of expression data for each cancer. Then, we proposed Similarity Kernel Fusion (SKF) to integrate the three kernels into one combined kernel. Finally, we used spectral clustering on integrated kernel to discover cancer subtypes.

We used an evaluation criteria (*P*-value) and a verification method (survival analysis) to evaluate the performance of SKF for the discovery of cancer subtypes. We compared SKF with single kernel and two kernel fusion strategies (SNF and UMKL) in three datasets. Results showed that SKF obtains a significant level of performance on *P*-value, and the survival curve of the subtypes was consistent with the clinical data. It demonstrates that SKF is an accurate computational tool for guiding clinical treatment.

Our method also has some limitations that require some attention. Since spectral clustering is a widely used and accepted cluster method, we are attempting to find an improved method to discover cancer subtypes more accurately. We will consider various machine learning methods and constructing kernel methods to predict cancer subtypes (Zeng et al., 2017; Ding et al., 2018; Zhang et al., 2018a,b,c; Zou et al., 2018). We also consider the potential possibility of developing computational models for cancer subtype identification based on microRNA information (Chen and Huang, 2017; Chen et al., 2017, 2018a,b; Hu et al., 2018).

## Data Availability Statement

The results and codes for this study can be found at the following address: https://github.com/guofei-tju/Cancer-subtypes.

## Author Contributions

FG and LJ conceived and designed the experiments. LJ and YX performed the experiments and analyzed the data. FG and YX wrote the paper. FG, YD, and JT supervised the experiments and reviewed the manuscript.

### Conflict of Interest Statement

The authors declare that the research was conducted in the absence of any commercial or financial relationships that could be construed as a potential conflict of interest.

## References

[B1] Brunet J. P. Tamayo P. Golub T. R. Mesirov J. P. (2004). Metagenes and molecular pattern discovery using matrix factorization. Proc. Natl. Acad. Sci. U.S.A. 101, 4164–4169. 10.1073/pnas.0308531101 15016911 PMC384712

[B2] Chen X. Huang L. (2017). LRSSLMDA: laplacian regularized sparse subspace learning for miRNA-disease association prediction. PLoS Comput. Biol. 13:e1005912. 10.1371/journal.pcbi.1005912 29253885 PMC5749861

[B3] Chen X. Huang L. Xie D. Zhao Q. (2018a). EGBMMDA: extreme gradient boosting machine for miRNA-disease association prediction. Cell Death Dis. 9:3. 10.1038/s41419-017-0003-x 29305594 PMC5849212

[B4] Chen X. Xie D. Wang L. Zhao Q. You Z. Liu H. (2018b). BNPMDA: bipartite network projection for miRNA–disease association prediction. Bioinformatics 34, 3178–3186. 10.1093/bioinformatics/bty333 29701758

[B5] Chen X. Xie D. Zhao Q. You Z. H. (2017). MicroRNAs and complex diseases: from experimental results to computational models. Brief. Bioinform. 18:558 10.1093/bib/bbx130 27345524 PMC5862301

[B6] Ding Y. Tang J. Guo F. (2017a). Identification of drug-target interactions via multiple information integration. Inform. Sci. 418, 546–560. 10.1016/j.ins.2017.08.045

[B7] Ding Y. Tang J. Guo F. (2017b). Identification of protein-ligand binding sites by sequence information and ensemble classifier. J. Chem. Inform. Model. 57, 3149–3161. 10.1021/acs.jcim.7b00307 29125297

[B8] Ding Y. Tang J. Guo F. (2018). Identification of drug-side effect association via multiple information integration with centered kernel alignment. Neurocomputing. 10.1016/j.neucom.2018.10.028

[B9] Finnegan T. J. Carey L. A. (2007). Gene-expression analysis and the basal-like breast cancer subtype. Future Oncol. 3, 55–63. 10.2217/14796694.3.1.55 17280502

[B10] Gao Y. Church G. (2005). Improving molecular cancer class discovery through sparse non-negative matrix factorization. Bioinformatics 21:3970–3975. 10.1093/bioinformatics/bti653 16244221

[B11] Ge S. G. Xia J. Sha W. Zheng C. H. (2017). Cancer subtype discovery based on integrative model of multigenomic data. IEEE ACM Trans. Comput. Biol. Bioinform. 14, 1115–1121. 10.1109/TCBB.2016.2621769 28113782

[B12] Guo F. Wang D. Wang L. (2018). Progressive approach for snp calling and haplotype assembly using single molecular sequencing data. Bioinformatics 34, 2012–2018. 10.1093/bioinformatics/bty059 29474523

[B13] Guo Y. Qi Y. Li Z. Shang X. (2018). Improvement of cancer subtype prediction by incorporating transcriptome expression data and heterogeneous biological networks, in Genome Informatics Workshop, (Kunming). 10.1186/s12920-018-0435-x PMC6311915 30598111

[B14] Hu H. Zhang L. Ai H. Zhang H. Fan Y. Zhao Q.. (2018). HLPI-ensemble: prediction of human lncRNA-protein interactions based on ensemble strategy. RNA Biol. 15:1. 10.1080/15476286.2018.1457935 29583068 PMC6152435

[B15] Katarzyna T. Patrycja C. Maciej W. (2015). The cancer genome atlas (TCGA): an immeasurable source of knowledge. Contemp. Oncol. 19, 68–77. 10.5114/wo.2014.47136 PMC4322527 25691825

[B16] Kruijf E. M. Engels C. C. van de Water W. Bastiaannet E. Smit V. T. van de Velde C. J.. (2013). Tumor immune subtypes distinguish tumor subclasses with clinical implications in breast cancer patients. Breast Cancer Res. Treat. 142, 355–364. 10.1007/s10549-013-2752-2 24197659

[B17] Le Van T. van Leeuwen M. Carolina Fierro A. De Maeyer D. Van den Eynden J. Verbeke L.. (2016). Simultaneous discovery of cancer subtypes and subtype features by molecular data integration. Bioinformatics 32:i445. 10.1093/bioinformatics/btw434 27587661

[B18] Liu X. Chu K. M. (2014). E-cadherin and gastric cancer: cause, consequence, and applications. Biomed. Res. Int. 2014:637308. 10.1155/2014/637308 25184143 PMC4145387

[B19] Luxburg U. V. (2007). A tutorial on spectral clustering. Stat. Comput. 17, 395–416. 10.1007/s11222-007-9033-z

[B20] Ma T. Zhang A. (2017). Integrate multi-omic data using affinity network fusion (anf) for cancer patient clustering, in IEEE International Conference on Bioinformatics and Biomedicine, (Kansas City, MO) 398–403. 29807109

[B21] Mager D. L. (2006). Bacteria and cancer: cause, coincidence or cure. J. Trans. Med. 4, 1–18. 10.1186/1479-5876-4-14 PMC1479838 16566840

[B22] Mariette J. Villavialaneix N. (2018). Unsupervised multiple kernel learning for heterogeneous data integration. Bioinformatics 34, 1009–1015. 10.1093/bioinformatics/btx682 29077792

[B23] Ng A. Y. Jordan M. I. Weiss Y. (2001). On spectral clustering: analysis and an algorithm, in International Conference on Neural Information Processing Systems: Natural and Synthetic, (Vancouver, BC) 849–856.

[B24] Nguyen D. V. Rocke D. M. (2002). Multi-class cancer classification via partial least squares with gene expression profiles. Bioinformatics 18, 1216–1226. 10.1093/bioinformatics/18.9.1216 12217913

[B25] Pan G. Jiang L. Tang J. Guo F. (2018). A novel computational method for detecting DNA methylation sites with DNA sequence information and physicochemical properties. Int. J. Mol. Sci. 19:511. 10.3390/ijms19020511 29419752 PMC5855733

[B26] Pan G. Tang J. Guo F. (2017). Analysis of co-associated transcription factors via ordered adjacency differences on motif distribution. Sci. Rep. 7:43597. 10.1038/srep43597 28240320 PMC5327392

[B27] Prat A. Pineda E. Adamo B. Galvn P. Fernndez A. Gaba L.. (2015). Clinical implications of the intrinsic molecular subtypes of breast cancer. Breast 24(Suppl. 2), S26–S35. 10.1016/j.breast.2015.07.008 26253814

[B28] Ren X. Fu H. Jin Q. (2015). Integrating heterogeneous genomic data to accurately identify disease subtypes. BMC Med. Genomics 8:78. 10.1186/s12920-015-0154-5 26589589 PMC4653838

[B29] Shen R. Olshen A. B. Ladanyi M. (2009). Integrative clustering of multiple genomic data types using a joint latent variable model with application to breast and lung cancer subtype analysis. Bioinformatics 25:2906. 10.1093/bioinformatics/btp543 19759197 PMC2800366

[B30] Song J. Tang J. Guo F. (2018). Identification of inhibitors of mmps enzymes via a novel computational approach:. Int. J. Biol. Sci. 14, 863–871. 10.7150/ijbs.24588 29989088 PMC6036742

[B31] Speicher N. K. Pfeifer N. (2015). Integrating different data types by regularized unsupervised multiple kernel learning with application to cancer subtype discovery. Bioinformatics 31, i268–i275. 10.1093/bioinformatics/btv244 26072491 PMC4765854

[B32] Teschendorff A. E. Miremadi A. Pinder S. E. Ellis I. O. Caldas C. (2007). An immune response gene expression module identifies a good prognosis subtype in estrogen receptor negative breast cancer. Genome Biol. 8:R157. 10.1186/gb-2007-8-8-r157 17683518 PMC2374988

[B33] Thanki K. Nicholls M. E. Gajjar A. Senagore A. J. Qiu S. Szabo C.. (2017). Consensus molecular subtypes of colorectal cancer and their clinical implications. Int. Biol. Biomed. J. 3, 105–111. 28825047 PMC5557054

[B34] Wang B. Mezlini A. M. Demir F. Fiume M. Tu Z. Brudno M.. (2014). Similarity network fusion for aggregating data types on a genomic scale. Nat. Methods 11, 333–337. 10.1038/nmeth.2810 24464287

[B35] Wei L. Luan S. Nagai L. A. E. Su R. Zou Q. (2018a). Exploring sequence-based features for the improved prediction of DNA n4-methylcytosine sites in multiple species. Bioinformatics. [Epub ahead of print]. 10.1093/bioinformatics/bty824 30239627

[B36] Wei L. Xing P. Shi G. Ji Z.-L. Zou Q. (2017). Fast prediction of protein methylation sites using a sequence-based feature selection technique. IEEE/ACM Trans. Comput. Biol. Bioinform. 34, 4007–4016 10.1109/TCBB.2017.2670558 28222000

[B37] Wei L. Zhou C. Chen H. Song J. Su R. (2018b). ACPred-FL: a sequence-based predictor based on effective feature representation to improve the prediction of anti-cancer peptides. Bioinformatics 34, 4007–4016. 10.1093/bioinformatics/bty451 29868903 PMC6247924

[B38] Wong G. Leckie C. Kowalczyk A. (2012). FSR: feature set reduction for scalable and accurate multi-class cancer subtype classification based on copy number. Bioinformatics 28:151. 10.1093/bioinformatics/btr644 22110244

[B39] Xu T. Le T. D. Liu L. Su N. Wang R. Sun B.. (2017). Cancersubtypes: an r/bioconductor package for molecular cancer subtype identification, validation and visualization. Bioinformatics 33, 3131–3133. 10.1093/bioinformatics/btx378 28605519

[B40] Xu T. Le T. D. Liu L. Wang R. Sun B. Li J. (2016). Identifying cancer subtypes from miRNA-tf-mRNA regulatory networks and expression data. PLoS ONE 11:e0152792. 10.1371/journal.pone.0152792 27035433 PMC4818025

[B41] Zeng X. Ding N. Rodrguez-Patn A. Quan Z. (2017). Probability-based collaborative filtering model for predicting gene–disease associations. BMC Med. Genomics 10(Suppl. 5):76. 10.1186/s12920-017-0313-y 29297351 PMC5751590

[B42] Zeng X. Liao Y. Liu Y. Zou Q. (2016). Prediction and validation of disease genes using hetesim scores. IEEE ACM Trans. Comput. Biol. Bioinform. 14, 687–695. 10.1109/TCBB.2016.2520947 26890920

[B43] Zhang W. Feng H. Wu H. Zheng X. (2017). Accounting for tumor purity improves cancer subtype classification from DNA methylation data. Bioinformatics 33:2651. 10.1093/bioinformatics/btx303 28472248 PMC6410888

[B44] Zhang W. Liu X. Chen Y. Wu W. Wang W. Li X. (2018a). Feature-derived graph regularized matrix factorization for predicting drug side effects. Neurocomputing 287, 154–162. 10.1016/j.neucom.2018.01.085

[B45] Zhang W. Yang W. Lu X. Huang F. Luo F. (2018b). The bi-direction similarity integration method for predicting microbe-disease associations. IEEE Access 6, 38052–38061. 10.1109/ACCESS.2018.2851751

[B46] Zhang W. Yue X. Lin W. Wu W. Liu R. Huang F. Liu F. (2018c). Predicting drug-disease associations by using similarity constrained matrix factorization. BMC Bioinform. 19:233. 10.1186/s12859-018-2220-4 29914348 PMC6006580

[B47] Zhao Q. Yu H. Ming Z. Hu H. Ren G. Liu H. (2018a). The bipartite network projection-recommended algorithm for predicting long non-coding RNA-protein interactions. Mol. Ther. Nucleic Acids 13, 464–471. 10.1016/j.omtn.2018.09.020 30388620 PMC6205413

[B48] Zhao Q. Zhang Y. Hu H. Ren G. Zhang W. Liu H. (2018b). Irwnrlpi: integrating random walk and neighborhood regularized logistic matrix factorization for lncRNA-protein interaction prediction. Front. Genet. 9:239. 10.3389/fgene.2018.00239 30023002 PMC6040094

[B49] Zou Q. Lin G. Jiang X. Liu X. Zeng X. (2018). Sequence clustering in bioinformatics: an empirical study. Brief. Bioinform. [Epub ahead of print]. 10.1093/bib/bby090 30239587

